# Grade-Related Differential Item Functioning in General English Proficiency Test-Kids Listening

**DOI:** 10.3389/fpsyg.2021.767244

**Published:** 2021-11-25

**Authors:** Linyu Liao, Don Yao

**Affiliations:** Department of English, University of Macau, Macau SAR, China

**Keywords:** grade, DIF, GEPT-Kids, listening, mixed-methods approach

## Abstract

Differential Item Functioning (DIF) analysis is always an indispensable methodology for detecting item and test bias in the arena of language testing. This study investigated grade-related DIF in the General English Proficiency Test-Kids (GEPT-Kids) listening section. Quantitative data were test scores collected from 791 test takers (Grade 5 = 398; Grade 6 = 393) from eight Chinese-speaking cities, and qualitative data were expert judgments collected from two primary school English teachers in Guangdong province. Two R packages “difR” and “difNLR” were used to perform five types of DIF analysis (two-parameter item response theory [2PL IRT] based Lord’s chi-square and Raju’s area tests, Mantel-Haenszel [MH], logistic regression [LR], and nonlinear regression [NLR] DIF methods) on the test scores, which altogether identified 16 DIF items. ShinyItemAnalysis package was employed to draw item characteristic curves (ICCs) for the 16 items in RStudio, which presented four different types of DIF effect. Besides, two experts identified reasons or sources for the DIF effect of four items. The study, therefore, may shed some light on the sustainable development of test fairness in the field of language testing: methodologically, a mixed-methods sequential explanatory design was adopted to guide further test fairness research using flexible methods to achieve research purposes; practically, the result indicates that DIF analysis does not necessarily imply bias. Instead, it only serves as an alarm that calls test developers’ attention to further examine the appropriateness of test items.

## Introduction

It is self-evident that language tests should be fair to all the test takers, rather than favoring or disfavoring any test taker groups because of construct-irrelevant issues such as gender, age, and native languages. This, however, cannot always be guaranteed no matter how carefully tests are designed. Conscientious test developers are expected to provide research evidence for the quality of their tests including the absence of bias (Kunnan, 2017). A well-known method to address this problem is the Differential Item Functioning (DIF) analysis, which examines whether test items function differentially toward two testing groups after controlling for the ability level of the groups (Holland and Wainer, 1993). Numerous scholars and test standards have advocated this method for the purpose of detecting construct-irrelevant and biased test items and improving test validity and fairness (e.g., Camilli and Shepard, 1994; Kunnan, 1997, 2000, 2004, 2017; Xi, 2010; Martinková and Drabinová, 2018).

Empirical studies have conducted DIF analysis to detect problematic test items, providing evidence for test quality and fairness. Existing studies, however, have mainly focused on the DIF effect toward testing groups classified by native languages (Abbott, 2007), gender (Aryadoust et al., 2011; Grover and Ercikan, 2017), and age (Geranpayeh and Kunnan, 2007; Aryadoust, 2012; Banerjee and Papageorgiou, 2016; Oliver et al., 2018). It is commonly known that learners of the same age may be placed into different grades, meaning that their years of English learning are different. But the grade-related DIF, emphasizing years of receiving English education, in tests for young children has been under-researched. The grade DIF usually occurs due to the discrepancy of grade levels. In other words, since higher grade students tend to be more cognitively developed due to their extra years of receiving English education, it is speculated that they are likely to be favored in a test even when the overall ability of the higher and the lower grade students is controlled for (i.e., they are more likely to get the correct answer even if they have the same overall ability as the lower grade students). Therefore, the indispensability of grade cannot be neglected in that it might influence test takers’ test performance, and further challenge the fairness and validity of the assessment.

While DIF items can be easily detected due to the development of statistical methods and software, relevant studies have rarely provided sound explanations for the existence of DIF. Geranpayeh and Kunnan (2007) reported that existing studies mainly focus on detecting DIF items, rather than identifying DIF sources. Similarly, Zumbo (2007) also pointed out this problem and called for exploring the reasons for DIF. In fact, some studies have made efforts to answer this *why* question (e.g., Li et al., 2004; Yao and Chen, 2020). However, only a weak relationship was found between gender DIF and task types, but no convincing explanations could be provided (see detailed discussion in the next section).

Considering the above-mentioned reasons, this study aims to detect grade DIF in a test for children, the GEPT-Kids (listening section^1^), and to find out potential reasons for such DIF. The GEPT-Kids claims that it is designed for primary school students but does not specify for which grade (Language Training and Testing Center, LTTC, 2015; Kunnan and Liao, 2019). Hence, it ought to be fair to all the pupils, rather than functioning differentially toward different grades. However, this has not been supported by empirical research evidence, which will be addressed in the current study.

## Literature Review

On account of the development of methods, empirical DIF studies have a relatively long history. Geranpayeh and Kunnan (2007) listed relevant studies in language testing from 1980 to 2005. Later, Kunnan (2017) continued to summarize DIF studies conducted from 1980 to 2017. Table 1 updates Kunnan’s (ibid) list by including more studies in the arena of language assessment during the same period. As the table shows, most of the existing DIF studies have placed the foci on L1 language, gender, age, and academic major. None of them have examined grade DIF, which, as argued in the introduction, is an influential factor for children due to years of English learning. In addition, tests for young children tend to be ignored in studies of this type. In terms of analytical methods, few of the existing studies used multiple methods. Therefore, their results may not be as precise or comprehensive as expected.

**TABLE 1:** DIF studies in language testing (1980–2017).

Author(s) and Year of Study	Specific Focus/Foci
Swinton and Powers (1980)	L1 language
Alderman and Holland (1981)	L1 language
Shohamy (1984)	Test method
Alderson and Urquhart (1985)	Academic major
Chen and Henning (1985)	L1 language
Zeidner (1986, 1987)	Gender and minorities
Hale (1988)	Major field and test content
Oltman et al. (1988)	L1 language
Kunnan (1990; 1992; 1995)	L1 language and gender
Sasaki (1991)	L1 language
Schohamy and Inbar (1991)	Question type and listening
Ryan and Bachman (1992)	Gender
Brown (1993)	Tape-mediated test
Ginther and Stevens (1998)	L1 language and ethnicity
Norton and Stein (1998)	Text content
Brown (1999)	L1 language
Takala and Kaftandjieva (2000)	L1 language
Lowenberg (2000)	Different Englishes
Kim (2001)	L1 language
Pae (2004)	Academic major and gender
Pae and Park (2006)*	Gender
Abbott (2007)*	L1 language
Ockey (2007)	L1 language
Roever (2007)	L1 language
Geranpayeh and Kunnan (2007)	Age
Allalouf and Abramzon (2008)*	L1 language
Kim and Jang (2009)	L1 language
Aryadoust et al. (2011)	Gender
Aryadoust (2012)*	Age
Harding (2012)	L1 language
Banerjee and Papageorgiou (2016)	Age
Grover and Ercikan (2017)*	Gender and socioeconomic status

*Items without an asterisk are cited from Kunnan (2017); items with an asterisk are added for this paper.*

Another deficiency with existing studies is that they can hardly give convincing explanations for the DIF effect that they found. At the early stage, Angoff (1993) concluded that DIF results are often confounding because it cannot be explained the reasons that some perfectly reasonable items are flagged. This question is still not well solved today, even though researchers have tried to explain DIF items. There are mainly two approaches to investigating DIF reasons: exploratory and confirmatory approaches. The exploratory approach detects DIF items first with statistical methods and then asks content experts to look for possible reasons for the DIF effect. Geranpayeh and Kunnan (2007) adopted this approach and proposed some reasons for DIF (e.g., the multidimensionality of test items). However, the reliability of explanations based on content experts’ experience and speculation sometimes is in doubt. The confirmatory approach is theory-based and hypothesis-driven. It first proposes hypotheses based on relevant literature, then detects DIF items, and finally checks the correctness of the hypotheses. For example, Li et al. (2004) attempted to find out the relationship between test items characteristics and gender DIF by coding test items based on Ibarra’s (2001) multi-context theory and Gallagher’s (1998) cognitive structure analysis approach. Correlation analyses between coded DIF index and detected DIF index suggested that multi-context theory-based approach could predict gender DIF more effectively (cf. Li et al., 2004). While this study shows that test items with certain characteristics tend to favor a particular group of test takers, and it inherently does not explain why such a relationship exists or why some test items with the same characteristics do not favor any group. Potential DIF sources are fruitful and reasons for DIF may vary across items. The confirmatory approach, however, tends to confine its focus to a particular reason and overgeneralizes this reason to all the items.

By contrast, the exploratory approach is open to any reasonable explanations and treats each question as a unique item with its own DIF sources. Therefore, the exploratory approach is considered to be more appropriate in exploring DIF sources in the current study. Since expert judgment alone does not give reliable reasons, Ercikan et al. (2010) used verbal report protocols of test takers to confirm DIF sources identified by experts. Such triangulation is supposed to improve the reliability of the study.

In view of the research gaps identified above and the merits and demerits of different research methods, the current study aims to examine grade-related DIF in the GEPT-Kids (listening section) through different analytical methods and explore the potential DIF sources through expert judgment and post-test interviews. To achieve these purposes, the following research questions are raised to guide the research.

(1) Are there any items in GEPT-Kids Listening exhibiting DIF toward different grade groups (Grade 5 and Grade 6)?
(2) What are the possible reasons for the detected grade DIF?

## Methods

To address the research questions, a mixed-methods sequential explanatory design was adopted in this study with two phases (Creswell et al., 2003; Ivankova et al., 2006). A quantitative DIF analysis was conducted to identify potential grade DIF items in GEPT-Kids listening (phase I). Then, qualitative expert judgment was conducted to find out the potential reasons for the detected grade DIF (phase II).

### Data Collection

Quantitative data used in this study were 791 pupils’ test scores in a GEPT-Kids listening test. The 791 participants (Grade 5 = 398; Grade 6 = 393) were English as a Foreign Language (EFL) learners from eight Chinese-speaking cities (i.e., Beijing, Shanghai, Guangzhou, Hong Kong, Macau, Taipei, Taichung, and Kaohsiung). The number of male (*N* = 399) and female (*N* = 392) students is similar. All the participants started learning English in Grade 1, and their first language is Chinese and second language is English. The GEPT-Kids listening test paper included four parts and 25 multiple-choice questions in total, with each item carrying one point. The test aims to test pupils’ understanding of common words, phrases, and simple sentences used in familiar topics that pupils may encounter in their daily life and school context (LTTC, 2015). The data were collected in a GEPT-Kids related project funded by LTTC^2^ in 2016–2017. The research assistants in this project went to the above-mentioned cities and administered the test in a Grade 5 and a Grade 6 class in each city. Then, the test papers were scored, and the results were input. The current study has gained permission from the LTTC to use relevant data.

Qualitative data were gained from expert judgment by two primary school English teachers in China. They both passed the Test for English Majors-Band 8 (TEM-8) and have been teaching English in the primary school in Guangdong province for more than ten years. Additionally, they are experienced in developing and designing tests for pupils. Firstly, the two teachers were asked to read the whole test paper carefully and judge whether certain items may favor Grade 5 and Grade 6 students and why. Then, they were informed which items were flagged as DIF items and tried to find out potential reasons. The two teachers sent their thoughts and opinions to the researchers during their analysis.

### Data Analysis

The quantitative data (i.e., test result) were analyzed by the Statistical Package for the Social Sciences (SPSS) v.24.0 and the RStudio. Firstly, SPSS v.24.0 was used to conduct a descriptive analysis of the test result. Mean and point-biserial correlation coefficients (item discrimination) were calculated to present an overview of the test result. Then, RStudio v.3.6.1 (R Studio Team, 2019) was used to conduct DIF analyses. Even though many DIF methods are available, different methods show different results and consensus has not been achieved on which method is the best. A feasible solution is to use different methods and examine all the results, in which way we may get closer to the truth that we are looking for Zumbo (2007); Zumbo et al. (2015). Also, even though various software has been developed for those DIF methods, such as the simultaneous item bias test (SIBTEST, Shealy and Stout, 1993), Differential Item Functioning Analysis System (DIFAS, Penfield, 2005, 2013), BILOG-MG (Zimowski, 1998), Item Response Theory for Patient-reported Outcomes (IRTPRO, Cai et al., 2011), not all of them are free, and more essentially, able to apply different methods [see Liu et al. (2019) published on *Frontiers in Psychology* for specific DIF methods discussion]. In terms of accessibility and flexibility, RStudio is an ideal tool, which is open to the public and allows the installation of various R packages to perform different kinds of analysis. Therefore, RStudio was chosen to conduct multiple DIF analyses. In RStudio, two R packages “difR” (Magis et al., 2010) and “difNLR” (Drabinová and Martinková, 2017; Hladka and Martinkova, 2020) were used to perform five types of DIF analysis (two-parameter [2PL] IRT based Lord’s chi-square and Raju’s area tests, MH, LR, and NLR DIF methods) on the test result. As different methods may flag different DIF items, the above-mentioned methods were taken to make the analysis as exhaustive as possible. Besides, ShinyItemAnalysis package (Martinková and Drabinová, 2018) was used to display item characteristic curves (ICCs) in RStudio to give a direct visual presentation about which test group the DIF items favor. Default settings were used for all the DIF analyses, interested readers may consult the manuals of related R packages online.

In the DIF analyses, it was hypothesized that Grade 6 students might be favored while Grade 5 students might be disfavored. The rationale is that for young children, grades are very likely to influence their test performance because different grade students vary in learning curriculums, psychological status, and other aspects. Even when the overall levels of different grade students are controlled for, Grade 6 students still tend to be favored (i.e., more likely to get the correct answer) because they are more cognitively developed, having larger working memory capacity and better comprehension of language, gaining more exposure to English and tests, and having been learning English for one more year. Since all of these developmental features seem to advantage higher grade students, in this study, Grade 6 was set as the reference group and Grade 5 as the focal group (the group at the risk of being disfavored, see Jiao and Chen, 2014).

The qualitative data were the two teachers’ self-reported thoughts during the analysis of the test paper. Their thoughts were summarized and reported below.

## Results

### Descriptive Statistics

Table 2 presents the descriptive statistics of the test performance data, which show the item difficulty and item discrimination. The mean of test items ranges from 0.52 to 0.98 for Grade 5 and from 0.65 to 0.98 for Grade 6. The highest mean for both Grade 5 and Grade 6 students lies in L9 and L12, and the lowest mean lies in L21. This result implies that students from both performed well on items L9 and L12, but poorly on item L21. The mean of most items is above 0.80, indicating that the test might be too easy for the test takers. In terms of item discrimination, most items have a discrimination level above 0.30 (McCowan and McCowan, 1999), indicating that they can discriminate test takers to some extent. To be more specific, high scorers are more likely to answer the items correctly, while low scorers are more likely to answer the items incorrectly. However, five items (L1, L3, L4, L5, and L9) may not be able to discriminate test takers at different levels because their corrected item-total correlation is too low (<0.30, McCowan and McCowan, 1999).

**TABLE 2:** Descriptive analysis of test performance data.

Test items	Mean (Grade 5)	Mean (Grade 6)	Corrected item-total correlation (Grade 5)	Corrected item-total correlation (Grade 6)
L1	0.82	0.86	0.353	0.251
L2	0.90	0.89	0.373	0.472
L3	0.94	0.97	0.378	0.258
L4	0.95	0.93	0.240	0.420
L5	0.96	0.96	0.313	0.277
L6	0.84	0.84	0.549	0.655
L7	0.83	0.86	0.426	0.516
L8	0.81	0.87	0.454	0.339
L9	0.98	0.98	0.243	0.305
L10	0.87	0.93	0.425	0.384
L11	0.88	0.91	0.411	0.424
L12	0.98	0.98	0.330	0.436
L13	0.94	0.97	0.525	0.428
L14	0.96	0.97	0.360	0.404
L15	0.94	0.93	0.500	0.410
L16	0.97	0.97	0.396	0.406
L17	0.92	0.92	0.526	0.518
L18	0.79	0.86	0.385	0.433
L19	0.89	0.88	0.436	0.509
L20	0.85	0.93	0.562	0.522
L21	0.52	0.65	0.332	0.519
L22	0.65	0.74	0.618	0.493
L23	0.71	0.80	0.543	0.613
L24	0.79	0.87	0.372	0.418
L25	0.62	0.76	0.510	0.635

### Differential Item Functioning Results

Table 3 summarizes DIF items flagged by different methods. From the table, the nonlinear regression test is the most sensitive method, detecting the most DIF items (14 items, over half of the total 25 items). On the other hand, 2PL IRT based Lord’s chi-square test and MH test are the least sensitive methods, detecting the fewest DIF items (four items). Five methods altogether flagged 16 items (i.e., L2, L3, L4, L6, L7, L9, L13, L15, L16, L17, L19, L20, L21, L22, L23, and L25) with potential DIF effect, which represent 64% of the total items. Among which, item L15 was flagged as DIF for the most times (four times). All these results were significant at 0.05 level (*p* < 0.05) and the effect sizes for the flagged items were moderate or large.

**TABLE 3:** DIF items flagged by different methods.

Method(s)	Flagged items	No. of flagged items
2PL IRT based Lord’s chi-square test	L15, L17, L19, L22	4
2PL IRT based Raju’s area test	L6, L15, L17, L21, L22	5
MH test	L4, L19, L20, L25	4
LR test	L4, L6, L15, L19, L20, L21, L22, L25	8
NLR test	L2, L3, L4, L6, L7, L9, L13, L15, L16, L17, L19, L20, L23, L25	14

The ICCs of the flagged 16 items show details about their DIF effect, which is displayed in Table 4. According to the table, four items have little DIF effect. The little effect represents that the test items are detected as DIF items, but the effect is minimal or even negligible. Also, six items favor Grade 6 students; four items favor Grade 5 students; and two items have non-uniform DIF effect (i.e., favoring one group at lower ability level and favoring the other group at higher ability level). Due to the space limit, not all the ICCs can be presented here; only an example of each DIF type is presented below.

**TABLE 4:** Types of DIF effect.

DIF type		Items	No. of flagged items
Little effect		L6, L7, L16, L17	4
Uniform DIF	Favoring Grade 6	L3, L13, L20, L22, L23, L25	6
	Favoring Grade 5	L2, L4, L9, L19	4
Non-uniform DIF		L15, L21	2

**FIGURE 1:**
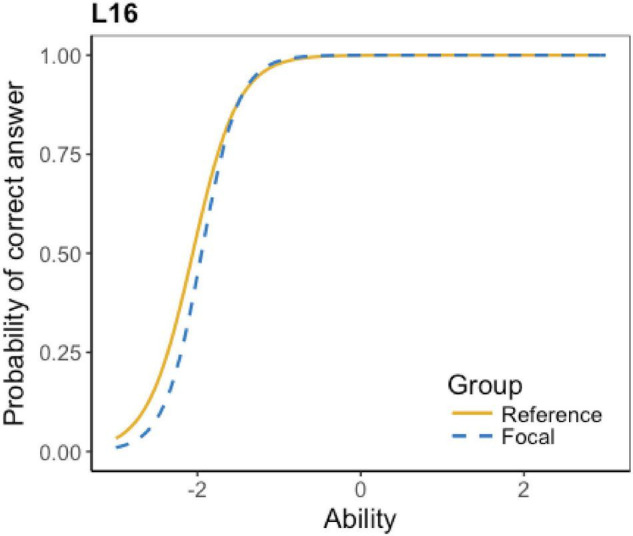
ICC of listening L16.

**FIGURE 2:**
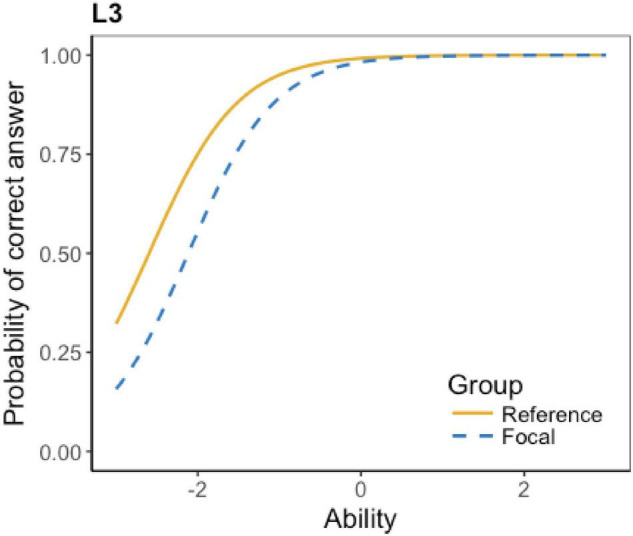
ICC of listening L3.

**FIGURE 3:**
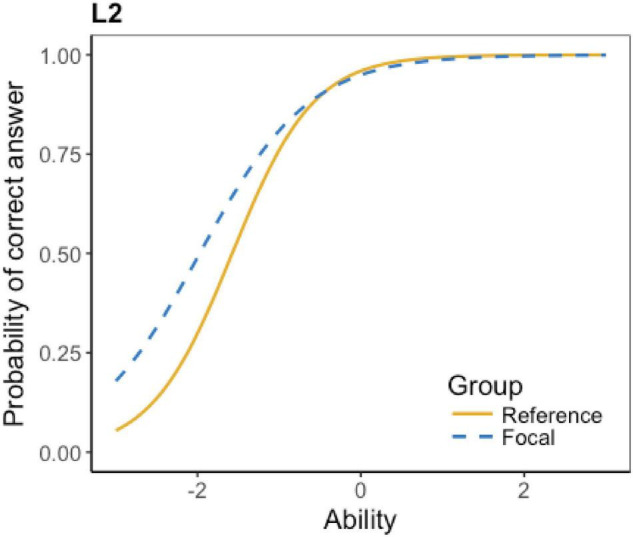
ICC of listening L2.

**FIGURE 4:**
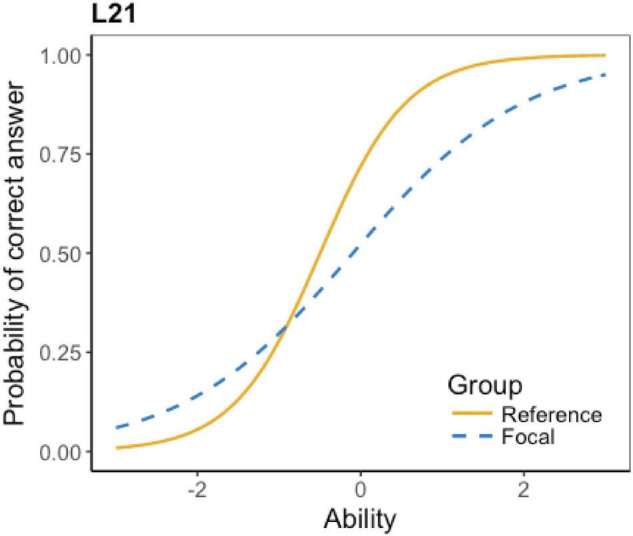
ICC of listening L21.

The ICCs show that the DIF effect of L6, L7, L16, and L17 is minimal. Taking L16 as an instance (see Figure 1), the two lines of the reference group and the focal group are almost identical, indicating that test takers at different levels have almost the same probability to get the correct answer. A striking feature is that most of the items, as predicted, favor Grade 6 students. As L3 in Figure 2 shows, at the lower ability level, the reference group (Grade 6) has a higher probability to get the correct answer. Surprisingly, there are four items favoring grade 5 students. For example, Figure 3 shows that at the lower ability level, the focal group (Grade 5) students are more likely to get the correct answer. Besides, L15 and L21 present a complex non-uniform DIF effect. As Figure 4 shows, at lower ability level, the focal group is more likely to get the correct answer, while at higher ability level, the reference group has a higher probability to get the correct answer.

### Expert Judgment

Expert judges were used to corroborate the findings from the DIF study. Without being told which items were identified in the DIF analysis, the two teachers who took part in the study could not identify any problems related to the grade factor with the test items. They did not think that any items would favor students in Grade 5 or Grade 6. After being told which items were flagged, they tried to come up with reasonable explanations for particular students being favored on certain items. They suggested that four items that may cause DIF: L20, L21, L22, and L25. For example, L21 asks *what is Nina going to do this evening?* with three options: A. See Dr. Li; B. Watch a ball game; C. Stay at home. The possible reason that they could think of is that *be going to* structure is not taught until the second term of Grade 5. Grade 5 students might have not learned this structure yet at the time of the data collection. Therefore, Grade 6 students might be favored on these four items with the *be going to* structure. Nevertheless, no plausible explanations could be given for other flagged items. For example, L2 requires test takers to look at a picture (a pair of socks) and judge whether what they hear (*This is a pair of socks.*) is the same as the picture. In the teachers’ opinion, test items like this one was considered fair to Grade 5 or Grade 6 students, because the topic and language were familiar to students in both grades, although these items were flagged as DIF items in the DIF analyses.

## Discussion and Conclusion

This study used a sequential explanatory mixed-methods design to detect DIF items in GEPT-Kids Listening. Different methods identified different potential DIF items, which may undermine the reliability of the analysis. Some researchers (e.g., Ferne and Rupp, 2007) recommend using multiple methods to validate DIF results. This study found that cross-validation may not be achieved through this way. Even though the five methods detected many potential DIF items, there were no items flagged by all of the five methods. The true value of using multiple DIF methods might be that they can detect potential DIF items to a larger extent and remind test developers to further examine those items. In addition, a potential limitation of research methods was that this study did not use correction for multiple comparisons in methods where DIF is tested item by item. Future DIF studies may try to use correction for multiple comparisons when they adopt multiple methods to conduct DIF analyses.

Similar to many of the previous studies (e.g., Uiterwijk and Vallen, 2005; Geranpayeh and Kunnan, 2007; Song et al., 2015), this study did not find convincing reasons for the DIF effect or any evidence of bias with the DIF items. It was suspected that L20, L21, L22, and L25 may favor Grade 6 because they contain *be going to* structure which is not taught until in the second term of Grade 5. This speculation makes sense to some extent. However, it does not explain why L21 exhibits non-uniform DIF, rather than favoring Grade 6 only. In addition, the two teachers’ speculation was solely based on their teaching experience in Guangdong province, so it may not be appropriate for this explanation to be generalized to other areas. Besides, there is no *golden standard*, i.e., criteria or rubrics, for teachers to appraise the suitability of test items in terms of grade. Moreover, even if the *be going to* structure is the reason for certain items favoring Grade 6, it does not mean that those items are biased, because language knowledge is a construct-relevant issue. The Grade 5 students have not learned this structure is not the problem with the test items themselves. In GEPT-Kids Listening, no evidence of bias could be found with the DIF items, which suggests that those items are not problematic. It is to be hoped that further research using expert judgment could consider containing more experts to share their opinions and feedback.

Even though this study detected many DIF items through statistical analyses, it should be noted that being flagged as DIF items does not necessarily mean that those items are biased (Angoff, 1993). Since DIF sources cannot be identified in this and many other studies, it might be reasonable to arrive at the conclusion that the result of DIF analysis only serves as an alarm that calls test developers’ attention to further examine the appropriateness of test items. When further examination cannot find evidence of bias with the test items, a verdict might be made that the test is free of bias. On the other hand, given the current study only lays emphasis on the grade factor, other variables such as gender and region are advocated to be included in further DIF research to present more comprehensive and representative research outcomes.

To sum up, the current study may both contribute to the test fairness to achieve its sustainable development: methodologically, robust mixed-methods research was adopted to guide further research using more flexible methods; practically, the study suggests test developers pay more attention to the test bias so that fairer test items could be developed, which may enhance the validity and reliability of the test and the bring about beneficial consequences to the educational system and practices or even the society.

## Data Availability Statement

The datasets presented in this article are not readily available because, due to the policy of LTTC, the data is not public. Requests to access the datasets should be directed to Rachel Wu, rwu@lttc.ntu.edu.tw.

## Ethics Statement

The studies involving human participants were reviewed and approved by the Language Training and Testing Center (LTTC). Written informed consent to participate in this study was provided by the participants’ legal guardian/next of kin.

## Author Contributions

LL was responsible for the manuscript draft. DY helped with data analyses and corresponding issues. Both authors contributed to the article and approved the submitted version.

## Conflict of Interest

The authors declare that the research was conducted in the absence of any commercial or financial relationships that could be construed as a potential conflict of interest.

## Publisher’s Note

All claims expressed in this article are solely those of the authors and do not necessarily represent those of their affiliated organizations, or those of the publisher, the editors and the reviewers. Any product that may be evaluated in this article, or claim that may be made by its manufacturer, is not guaranteed or endorsed by the publisher.

## Footnotes

1
https://www.geptkids.org.tw/

2
https://www.lttc.ntu.edu.tw/aboutthelttc.htm
